# Supplementary material for: Protocol for the development of the STrengthening the Reporting Of Pharmacogenetic Studies (STROPS) guideline: checklist of items for reporting pharmacogenetic studies
Source: BMJ Open. 2019 Jul 11;9(7):e030212. doi: 10.1136/bmjopen-2019-030212 (PMC6629424; doi:10.1136/bmjopen-2019-030212)
Supplement: Supplementary file 1 [file bmjopen-2019-030212supp001.pdf]

Supplementary file 1: Initial invitation e-mail

Dear NAME,

We are a team of researchers based in different institutions across the UK (University of Liverpool; University of St. Andrews; Cochrane Editorial Unit, London), and we are currently developing the STrengthening the Reporting Of Pharmacogenetic Studies (STROPS) guideline. We are conducting a Delphi survey to gain consensus opinion on which reporting items ought to be included in this guideline.

We would like to invite you to be part of the development of this guideline by completing our Delphi survey.

The Delphi survey will consist of two rounds of electronic-based survey, response and feedback. Participants will be asked to score reporting items based on their opinions about the importance of these items for inclusion in the reporting guideline. All scoring data will be anonymised. Participation is of course optional, and furthermore, completion of the first round survey does not necessitate completion in the second round survey. We will assume informed consent if we receive a response to the first round of the Delphi survey.

If you would like to participate in the development of this reporting guideline, please complete the first round of the Delphi survey by DATE using the following link: LINK

Kind Regards,
Marty Richardson

On behalf of the Steering Committee of the STROPS (STrengthening the Reporting Of Pharmacogenetic Studies) project
