# Supplementary material for: Protocol for the development of the STrengthening the Reporting Of Pharmacogenetic Studies (STROPS) guideline: checklist of items for reporting pharmacogenetic studies
Source: BMJ Open. 2019 Jul 11;9(7):e030212. doi: 10.1136/bmjopen-2019-030212 (PMC6629424; doi:10.1136/bmjopen-2019-030212)
Supplement: Author's manuscript [file bmjopen-2019-030212.draft_revisions.pdf]

BMJ Open is committed to open peer review. As part of this commitment we make the peer review history of every article we publish publicly available.

When an article is published we post the peer reviewers' comments and the authors' responses online. We also post the versions of the paper that were used during peer review. These are the versions that the peer review comments apply to.

The versions of the paper that follow are the versions that were submitted during the peer review process. They are not the versions of record or the final published versions. They should not be cited or distributed as the published version of this manuscript.

BMJ Open is an open access journal and the full, final, typeset and author-corrected version of record of the manuscript is available on our site with no access controls, subscription charges or pay-per-view fees (http://bmjopen.bmj.com).

If you have any questions on BMJ Open's open peer review process please email info.bmjopen@bmj.com

The STrengthening the Reporting Of Pharmacogenetic Studies (STROPS) guideline: checklist of items for reporting pharmacogenetic studies (PROTOCOL)

Journal:	*BMJ Open*
Manuscript ID	bmjopen-2019-030212
Article Type:	Protocol
Date Submitted by the Author:	04-Mar-2019

Complete List of Authors:	Richardson, Marty; University of Liverpool, Department of Biostatistics<br>Kirkham, Jamie; University of Liverpool, Department of Biostatistics<br>Dwan , Kerry M; Cochrane Editorial Unit<br>Sloan, Derek; University of Saint Andrews, School of Medicine<br>Davies, Geraint; University of Liverpool, Department of Clinical Infection, Microbiology and Immunology<br>Jorgensen, Andrea; University of Liverpool, Department of Biostatistics

Keywords:	Pharmacogenetics, Reporting guideline, Delphi survey

SCHOLARONE™
Manuscripts

The STrengthening the Reporting Of Pharmacogenetic Studies (STROPS) guideline: checklist of items for reporting pharmacogenetic studies (PROTOCOL)

Marty Richardson[1*] (ORCID iD: 0000-0002-7097-8704)

Jamie Kirkham[1]

Kerry Dwan[2]

Derek J Sloan[3]

Geraint Davies[4]

Andrea L Jorgensen[1]

[1] Department of Biostatistics, University of Liverpool, L69 3GB, UK

[2] Cochrane Editorial Unit, London, SW1Y 4QX, UK

[3] School of Medicine, University of St Andrews, KY16 9TF, UK

[4] Department of Clinical Infection, Microbiology and Immunology, University of Liverpool, L69 3GB, UK

* Corresponding author

Marty Richardson,

2nd floor,

Whelan Building,

University of Liverpool,

L69 3GB, UK

E-mail: mhr@liverpool.ac.uk

Telephone: (+44) 0151 795 7523

For peer review only

Word count: 2404

For peer review only

ABSTRACT

Introduction

Large sample sizes are often required to detect statistically significant associations between pharmacogenetic markers and treatment response. Meta-analysis may be performed to synthesise data from several studies, increasing sample size and consequently power to detect significant genetic effects. However, performing robust synthesis of data from pharmacogenetic studies is often challenging due to poor reporting of key data in study reports. There is currently no guideline for the reporting of pharmacogenetic studies. The aim of this project is to develop the STROPS (STrengthening the Reporting Of Pharmacogenetic Studies) guideline. The STROPS guideline will not only improve the reporting of pharmacogenetic studies, but will also facilitate the conduct of high-quality meta-analyses, and thus improve the power to detect genetic associations.

Methods and analysis

We will establish a preliminary checklist of reporting items to be considered for inclusion in the guideline. We will then conduct a Delphi survey of key stakeholder groups to gain consensus opinion on which reporting items to include in the final guideline. The Delphi survey will consist of two rounds: the first-round will invite participants to score items from the preliminary checklist and to suggest additional relevant items; the second-round will provide feedback from the previous round and invite participants to re-score the items. Following the second-round, we will summarise the distribution of scores for each item, stratified by stakeholder group.

The Steering Committee for the project will meet to consider the results of the Delphi survey and to finalise the list of reporting items. We will then draft, pilot-test and publish the STROPS (STrengthening the Reporting Of Pharmacogenetic Studies) reporting guideline and accompanying explanatory document.

For peer review only

Ethics and dissemination

The University of Liverpool Ethics Committee has confirmed ethical approval for this study (reference: 3586). Dissemination activities will include presenting the reporting guideline at conferences relevant to pharmacogenetics research.

ARTICLE SUMMARY

Strengths and limitations of this study

- We will conduct our project using methodology proposed by the EQUATOR (Enhancing the QUAlity and Transparency Of health Research) network for the development of reporting guidelines
- The Delphi survey will enable us to gain information about the opinions of a wide group of participants
- Our study design is limited by the fact that the consensus meeting will only involve the six members of the Steering Committee, as we are unable to fund travel and accommodation for representatives of the key stakeholder groups

For peer review only

INTRODUCTION

Pharmacogenetic studies investigate associations between genetic variants (SNPs) and treatment response for a particular drug of interest, in terms of both benefits (therapeutic effect) and harms (adverse effects). The aim of performing such studies is to identify ways that drug efficacy may be maximised, and that toxicity may be minimised. If a significant association between a genetic variant and a treatment response outcome is identified, patients may eventually be genotyped in clinical practice before being prescribed a certain treatment. The health care provider may then refer to the results of the genotyping test when determining whether to prescribe the drug, and if prescribed, the appropriate dosage of the drug. Such an approach is known as "personalised medicine".

Outcomes from pharmacogenetic studies are often likely to be complex traits; genetic influence may be explained by several genetic variants each having only a small effect on outcome. Consequently, large sample sizes are typically required to detect statistically significant associations between a genetic variant and treatment response. Meta-analysis improves sample size and consequently increases the power to detect significant genetic effects. However, significant differences are often observed between pharmacogenetic studies in terms of the genetic variants investigated, definition of genetic subgroups and outcomes, and assumptions made in the analyses for example about the underlying mode of inheritance. This can significantly reduce the number of studies available to contribute to a single meta-analysis. This problem is compounded by poor reporting of key data in study reports. The aim of our project is to develop a guideline for the reporting of pharmacogenetic studies and an explanatory document using methodology proposed by EQUATOR (Enhancing the QUAlity and Transparency Of health Research).[1] Such a guideline would facilitate the conduct of high-quality systematic reviews and meta-analyses, thus improving power to detect genetic associations.

For peer review only

An explanation of the terms used in this proposal

Steering Committee

The panel consisting of six members: Marty Richardson (researcher into meta-analysis of pharmacogenetic studies), Jamie Kirkham (researcher into consensus methodology and developer of reporting guidelines), Kerry Dwan (researcher into systematic review methodology), Derek Sloan (Senior Clinical Lecturer in Infectious Diseases), Gerry Davies (Professor of Infection Pharmacology) and Andrea Jorgensen (researcher into statistical methods for pharmacogenetics, including evidence synthesis methods).

Delphi participants

Participants in the Delphi survey.

METHODS AND ANALYSIS

We (the Steering Committee) will develop the guideline for pharmacogenetic studies in five stages:

1. Establish a preliminary checklist of reporting items to be considered for inclusion in the reporting guideline for pharmacogenetic studies (Stage 1).
2. Conduct a Delphi survey to gain consensus opinion on reporting items to be considered within a reporting guideline for pharmacogenetic studies (Stage 2).
3. Hold a Steering Committee meeting to consider the results of the Delphi survey and to finalise the list of items for the reporting guideline (Stage 3).
4. Develop and publish a high-quality reporting guideline and a detailed explanatory document (Stage 4).
5. Dissemination of the published guideline (Stage 5).

Stage 1: Preliminary checklist of reporting items

For peer review only

We will establish a preliminary checklist of reporting items by:

i. Including items from existing relevant guidelines: Existing relevant guidelines will be identified by considering all guidelines listed on the EQUATOR website,[2] under the clinical area of genetics. Two authors (MR and ALJ) will assess existing guidelines to be relevant if they are applicable to pharmacogenetics studies from their experience of reviewing and undertaking meta-analyses of pharmacogenetics studies. We will include items from these existing guidelines in our preliminary checklist if they ensure transparency of reporting of pharmacogenetic studies, and consequently will enable future evidence synthesis.
ii. Supplementing this list with any additional items thought to be important: Additional items will be identified by consideration of the quality assessment checklist for pharmacogenetic studies developed by Jorgensen and Williamson,[3] and through discussion amongst the Steering Committee.
iii. Providing an explanation and/or example of each reporting item: One author (MR) will draft the explanation and/or example for each reporting item. This will ensure that each reporting item is clear to participants in the Delphi survey.

All members of the Steering Committee will review and approve this preliminary list of reporting items and explanations/examples before the Delphi survey begins.

Stage 2: Delphi survey

Design

The Delphi process will consist of two rounds of electronic-based survey, response and feedback. The first round survey will include scoring of reporting guideline items from the preliminary list formed at Stage 1 and will invite additional items not included in this list. A second round survey will then be undertaken providing feedback from the previous round and inviting participants to re-score

For peer review only

these items. Any additional reporting items identified by participants in the first round will be included for scoring by participants in the second round of the Delphi process.

Participants

We will invite three groups of stakeholders to participate in the Delphi survey. Stakeholder groups will be chosen to encompass all aspects of pharmacogenetic research.

1. Those who undertake primary pharmacogenetic research

We will ask members of pharmacogenetics networks, such as the Pharmacogenomics Research Network (PGRN) and the UK Pharmacogenetics and Stratified Medicine Network, to participate in our Delphi survey. We will perform searches to identify these networks and contact experts in the field to ensure that all major networks across the globe are identified.

2. Those who systematically review pharmacogenetic research data

We will email the contact authors of systematic reviews of pharmacogenetics studies identified by searching PubMed, using appropriate search terms such as “pharmacogenetics”, “pharmacogenomics”, “systematic review” and “meta-analysis”. We will consult an information specialist (Eleanor Kotas) to design the search strategy). We will ask these authors to complete the survey if they participated in the data extraction and/or data synthesis of the review, and we will also ask this author to forward the survey on to other authors who participated in the data extraction and/or data synthesis.

3. Those who publish pharmacogenetic research

We will identify these individuals by contacting the editors-in-chief of key pharmacogenetics journals. We will ask the editors-in-chief to participate in the survey themselves, and also to forward the survey on to editors at their journal.

For peer review only

Our aim is to maximise the number of participants who complete the Delphi survey, to ensure that the number of participants is sufficiently large to yield a meaningful statistical analysis. We hope that the snowballing technique i.e. asking study authors and editors-in-chief to forward the survey on to other individuals, will ensure that number of participants is sufficiently large.

Recruitment process and ethical considerations

We will e-mail the individuals listed above with an outline of the project and an invitation to complete Round 1 of the Delphi survey within three weeks. We will inform invitees that participation in the survey is optional, and that we will assume informed consent if an invitee responds to the survey. We will inform invitees that all data will be anonymised; we will allocate a unique identification number to each participant in the Delphi survey.

We will send a reminder e-mail at the end of the second week to prompt completion of the survey. All participants who complete the first round of the Delphi survey will be invited to participate in the second round. However, we will inform invitees that completion in the first round does not necessitate completion in the second round, and we will remind participants of the first round that this is the case, when we invite them to complete the second round.

Participant characteristics

We will ask participants to provide their name and their consent to be acknowledged as a participant in the Delphi survey in publications arising from this project. Demographic data regarding the participant’s profession and previous involvement with reporting guideline development will be collected; all demographic data will be anonymised.

Delphi scoring

For peer review only

Participants will be asked to score each of the reporting guideline items listed using a scale of 1 to 9, with 1 to 3 labelled 'not important for inclusion in the guideline', 4 to 6 labelled 'important but not critical for inclusion in the guideline' and 7 to 9 labelled 'critical for inclusion into the guideline'.[4] Participants will also be given the option to score a reporting guideline item as 'unsure' if they are unable to offer an opinion as to whether the item is important or not.

Software

The Delphi survey will be conducted using DelphiManager, a web-based system designed by the COMET Initiative (http://www.comet-initiative.org/delphimanager/) to facilitate the building and management of Delphi surveys.

Delphi Round 1

Reporting guideline items will be presented in the order in which they would be addressed in the pharmacogenetic study report and will be grouped under relevant headings (i.e. title and abstract, introduction, methods, results, discussion and other information). Participants will be asked to score each item as described previously. Participants will also be given the chance to add items that they believe should be included in the reporting guideline.

Round 1 analysis

For each item, the number of participants who have scored the item and the distribution of scores will be summarised. We will review all additional guideline reporting items listed by participants to ensure that they are not covered by the existing list of reporting guideline items. Additional reporting guidelines items that are not already covered will undergo formal review and discussion by the Steering Committee, and if appropriate, will be added to the list of reporting guideline items presented in Round 2.

For peer review only

Delphi Round 2

In Round 2, each participant who participated in Round 1 will be shown the number of respondents and distribution of scores for each item from Round 1, for each stakeholder group separately. Participants will also be reminded how they personally scored each item in Round 1. Participants will be asked to consider the responses from other Delphi participants, and to re-score the items.

In addition, if a participant changes their score from ‘not critical’ in Round 1 to ‘critical’ in Round 2 or from ‘critical’ in Round 1 to ‘not critical’ in Round 2, they will be asked to provide their reasoning for this change.

Additional items identified as part of Round 1 will be scored by participants in Round 2.

Round 2 analysis

For each item, the number of respondents and the distribution of scores will be summarised. We will assess the possibility of attrition bias occurring by comparing item scores from participants who completed Round 1 only, to item scores from participants who completed both Round 1 and Round 2. We will also examine changes in scores between rounds and summarise the reasons given for changes from ‘critical’ to ‘non-critical’ and vice versa.

Consensus definition

Guideline reporting items will be prioritised if at least 70% of participants score them as ‘critical’.

Stage 3: Steering Committee meeting

The Steering Committee will meet face-to-face to consider the results of the Delphi survey and to finalise the list of items for the draft reporting guideline. At the meeting, MR will present a summary of the results of how each stakeholder group had scored each reporting guideline item (from Stage

For peer review only

2), and the number of stakeholder groups who achieved consensus. The Steering Committee will discuss each reporting guideline item in turn, and will make a decision on whether to include the item in the reporting guideline or not. Items that reached consensus from all stakeholder groups in the Delphi survey will be considered first. Each remaining item will then be considered in turn according to the number of stakeholder groups where consensus was achieved, i.e. the next batch of items to be considered will be those that reached consensus in all but one stakeholder group.

Stage 4: Development and publication of reporting guideline and explanatory document

We will draft the initial reporting guideline and the explanatory document concurrently. The purpose of the explanatory document is to provide the meaning and rationale for each reporting item alongside examples of good reporting practice. For each item, we will also document the origin of the item (Steering Committee or Delphi participants) and the degree of consensus achieved from the Delphi survey.

We will pilot-test the draft reporting guideline with researchers who are yet to publish the findings of their pharmacogenetics study, and with researchers who have already published a pharmacogenetics study. We will identify these individuals by contacting pharmacogenetics researchers based at the University of Liverpool, and by contacting authors of published pharmacogenetics studies listed on PubMed. We will incorporate feedback on the content, format, and usefulness of the guideline from these researchers in the final reporting guideline.

Stage 5: Dissemination of the reporting guideline

Dissemination activities will include presenting the final reporting guideline at conferences relevant to pharmacogenetics research.

For peer review only

PATIENT AND PUBLIC INVOLVEMENT STATEMENT

The STROPS guideline will be developed without patient involvement. Patients were not invited to contribute to the writing or editing of this document for readability or accuracy.

ETHICS

The University of Liverpool Ethics Committee has been consulted and confirmed ethical approval for this study (Reference: 3586).

DISCUSSION

We plan to conduct our project using robust methodology for developing reporting guidelines proposed by the EQUATOR network.[1] Using such methodology will ensure that the resulting reporting guideline is useful and widely disseminated. The EQUATOR approach includes a face-to-face consensus meeting, which follows the Delphi survey. This meeting often involves the steering group and a selection of stakeholders who took part in the Delphi survey. Simera et al.[5] conducted a survey of authors of 37 reporting guidelines and reported that the median number of people participating in consensus meetings for these reporting guidelines was 22. Due to a lack of funding for this project to cover travel and accommodation costs, we will be unable to arrange a consensus meeting including this number of participants. Therefore, our consensus meeting will only involve the members of the Steering Committee (n=6). We will invite a large, international and multidisciplinary cohort to participate in the Delphi survey, so that the Steering Committee are able to base their decisions on the opinions of this wider cohort.

There is currently no guideline for the reporting of pharmacogenetic studies that has been developed using a widely-accepted robust methodology. The final guideline will not only improve the transparency of reporting of pharmacogenetic studies, but will also facilitate the conduct of high-quality systematic reviews and meta-analyses, and thus improve the power to detect genetic

For peer review only

associations. With the increasing number of meta-analyses of pharmacogenetic studies that are being undertaken, it is important that reporting of key data in study reports is improved in order to allow robust synthesis of the studies.

REFERENCES

1. Moher D, Schulz KF, Simera I, et al. Guidance for developers of health research reporting guidelines. *PLoS Med* 2010;7(2):e1000217.
2. UK EQUATOR Centre. Equator Network 2018 [Available from: https://www.equator-network.org/ accessed 25th Feb 2019].
3. Jorgensen AL, Williamson PR. Methodological quality of pharmacogenetic studies: issues of concern. *Stat Med* 2008;27(30):6547-69.
4. Guyatt GH, Oxman AD, Kunz R, et al. GRADE guidelines: 2. Framing the question and deciding on important outcomes. *J Clin Epidemiol* 2011;64(4):395-400.
5. Simera I, Altman DG, Moher D, et al. Guidelines for reporting health research: the EQUATOR network's survey of guideline authors. *PLoS Med* 2008;5(6):e139.

AUTHOR STATEMENT

MR, ALJ, JK and KD conceived the idea for the project. All authors contributed to the design of the study. MR is the guarantor, and drafted the manuscript. All authors read, provided feedback and approved the final manuscript.

FUNDING

MR is supported partly by Liverpool Reviews and Implementation Group (LR*i*G, based at the University of Liverpool), based on funding from the National Institute for Health Research Health Technology Assessment Programme (URL, http://www.nets.nihr.ac.uk/programmes/hta), and partly by the Research, Evidence and Development Initiative (READ-It) project. READ-It (project number 300342-104) is funded by UK aid from the UK government; however, the views expressed do not necessarily reflect the UK government's official policies.

CONFLICTS OF INTERESTS

The authors declare that they have no conflicts of interests.

For peer review only

BMJ Open

Protocol for the development of the STrengthening the Reporting Of Pharmacogenetic Studies (STROPS) guideline: checklist of items for reporting pharmacogenetic studies

Journal:	*BMJ Open*
Manuscript ID	bmjopen-2019-030212.R1
Article Type:	Protocol
Date Submitted by the Author:	10-Jun-2019

Complete List of Authors:	Richardson, Marty; University of Liverpool, Department of Biostatistics<br>Kirkham, Jamie; University of Liverpool, Department of Biostatistics<br>Dwan , Kerry M; Cochrane Editorial Unit<br>Sloan, Derek; University of Saint Andrews, School of Medicine<br>Davies, Geraint; University of Liverpool, Department of Clinical Infection, Microbiology and Immunology<br>Jorgensen, Andrea; University of Liverpool, Department of Biostatistics

**Primary Subject Heading**:	Genetics and genomics
Secondary Subject Heading:	Genetics and genomics
Keywords:	Pharmacogenetics, Reporting guideline, Delphi survey

SCHOLARONE™
Manuscripts

Protocol for the development of the STrengthening the Reporting Of Pharmacogenetic Studies (STROPS) guideline: checklist of items for reporting pharmacogenetic studies

Marty Richardson[1*] (ORCID iD: 0000-0002-7097-8704)

Jamie Kirkham[1]

Kerry Dwan[2]

Derek J Sloan[3]

Geraint Davies[4]

Andrea L Jorgensen[1]

[1] Department of Biostatistics, University of Liverpool, L69 3GB, UK

[2] Cochrane Editorial Unit, London, SW1Y 4QX, UK

[3] School of Medicine, University of St Andrews, KY16 9TF, UK

[4] Department of Clinical Infection, Microbiology and Immunology, University of Liverpool, L69 3GB, UK

* Corresponding author

Marty Richardson,

2nd floor,

Whelan Building,

University of Liverpool,

L69 3GB, UK

E-mail: mhr@liverpool.ac.uk

Telephone: (+44) 0151 795 7523

Word count: 3163

For peer review only

ABSTRACT

Introduction

Large sample sizes are often required to detect statistically significant associations between pharmacogenetic markers and treatment response. Meta-analysis may be performed to synthesise data from several studies, increasing sample size and consequently power to detect significant genetic effects. However, performing robust synthesis of data from pharmacogenetic studies is often challenging due to poor reporting of key data in study reports. There is currently no guideline for the reporting of pharmacogenetic studies. The aim of this project is to develop the STROPS (STrengthening the Reporting Of Pharmacogenetic Studies) guideline. The STROPS guideline will facilitate the conduct of high-quality meta-analyses, and thus improve the power to detect genetic associations.

Methods and analysis

We will establish a preliminary checklist of reporting items to be considered for inclusion in the guideline. We will then conduct a Delphi survey of key stakeholder groups to gain consensus opinion on which reporting items to include in the final guideline. The Delphi survey will consist of two rounds: the first-round will invite participants to score items from the preliminary checklist and to suggest additional relevant items; the second-round will provide feedback from the previous round and invite participants to re-score the items. Following the second-round, we will summarise the distribution of scores for each item, stratified by stakeholder group.

The Steering Committee for the project and representatives from the key stakeholder groups will meet to consider the results of the Delphi survey and to finalise the list of reporting items. We will then draft, pilot-test and publish the STROPS (STrengthening the Reporting Of Pharmacogenetic Studies) reporting guideline and accompanying explanatory document.

For peer review only

Ethics and dissemination

The University of Liverpool Ethics Committee has confirmed ethical approval for this study (reference: 3586). Dissemination activities will include presenting the reporting guideline at conferences relevant to pharmacogenetics research.

ARTICLE SUMMARY

Strengths and limitations of this study

- We will conduct our project using methodology proposed by the EQUATOR (Enhancing the QUAlity and Transparency Of health Research) network for the development of reporting guidelines
- The Delphi survey will enable us to gain information about the opinions of a wide group of participants
- Our study design is limited by the fact that the consensus meeting will only involve the six members of the Steering Committee and one or two representatives from the key stakeholder groups, and will be conducted via conference call

For peer review only

INTRODUCTION

Pharmacogenetic studies investigate associations between genetic variants and treatment response for a particular drug of interest, in terms of both benefits (therapeutic effect) and harms (adverse effects). The aim of performing such studies is to identify ways that drug efficacy may be maximised, and that toxicity may be minimised. If a significant association between a genetic variant and a treatment response outcome is identified, patients may eventually be genotyped in clinical practice before being prescribed a certain treatment. The health care provider may then refer to the results of the genotyping test when determining whether to prescribe the drug, and if prescribed, the appropriate dosage of the drug. Such an approach is known as “personalised medicine”.

Outcomes from pharmacogenetic studies are often likely to be complex traits; genetic influence may be explained by several genetic variants each having only a small effect on outcome. Consequently, large sample sizes are typically required to detect statistically significant associations between a genetic variant and treatment response. Meta-analysis improves sample size and consequently increases the power to detect significant genetic effects. However, significant differences are often observed between pharmacogenetic studies in terms of the genetic variants investigated, definition of genetic subgroups and outcomes, and assumptions made in the analyses for example about the underlying mode of inheritance. This can significantly reduce the number of studies available to contribute to a single meta-analysis. This problem is compounded by poor reporting of key data in study reports. For example, if the authors of a particular study do not report the number of participants in each genotype group and outcomes for each genotype group separately, it may not be possible for researchers conducting a systematic review to include this study in a meta-analysis. Furthermore, lack of reporting of participants’ ethnic backgrounds can also severely hinder investigations of heterogeneity, which form a key part of any systematic review and/or meta-analysis. Genetic associations are likely to vary according to ethnicity; it is therefore recommended

For peer review only

that meta-analyses are always stratified by ethnicity, and pooling of results should only be performed if effect estimates for different ethnic groups appear sufficiently similar.[1]

The aim of our project is to develop a guideline for the reporting of pharmacogenetic studies and an explanatory document using methodology proposed by EQUATOR (Enhancing the QUAlity and Transparency Of health Research).[2] Such a guideline would facilitate the conduct of high-quality systematic reviews and meta-analyses, thus improving power to detect genetic associations.

An explanation of the terms used in this proposal

Steering Committee

The panel consisting of six members: Marty Richardson (researcher into meta-analysis of pharmacogenetic studies), Jamie Kirkham (researcher into consensus methodology and developer of reporting guidelines), Kerry Dwan (researcher into systematic review methodology), Derek Sloan (Senior Clinical Lecturer in Infectious Diseases), Gerry Davies (Professor of Infection Pharmacology) and Andrea Jorgensen (researcher into statistical methods for pharmacogenetics, including evidence synthesis methods).

Delphi participants

Participants in the Delphi survey.

METHODS AND ANALYSIS

We (the Steering Committee) will develop the guideline for pharmacogenetic studies in five stages:

1. Establish a preliminary checklist of reporting items to be considered for inclusion in the reporting guideline for pharmacogenetic studies (Stage 1).

For peer review only

2. Conduct a Delphi survey to gain consensus opinion on reporting items to be considered within a reporting guideline for pharmacogenetic studies (Stage 2).
3. Hold a consensus meeting to consider the results of the Delphi survey and to finalise the list of items for the reporting guideline (Stage 3).
4. Develop and publish a high-quality reporting guideline and a detailed explanatory document (Stage 4).
5. Dissemination activities to raise awareness of the published guideline, including presenting the guideline at relevant conferences (Stage 5).

Stage 1: Preliminary checklist of reporting items

We will establish a preliminary checklist of reporting items by:

i. Including items from existing relevant guidelines: Existing relevant guidelines will be identified by considering all guidelines listed on the EQUATOR website,[3] under the clinical area of genetics. Two authors (MR and ALJ) will assess existing guidelines to be relevant if they are applicable to pharmacogenetics studies from their experience of reviewing and undertaking meta-analyses of pharmacogenetics studies. We will include items from these existing guidelines in our preliminary checklist if they ensure transparency of reporting of pharmacogenetic studies, and consequently will enable future evidence synthesis.

ii. Supplementing this list with any additional items thought to be important: Additional items will be identified by consideration of the quality assessment checklist for pharmacogenetic studies developed by Jorgensen and Williamson,[4] and through discussion amongst the Steering Committee.

iii. Providing an explanation and/or example of each reporting item: One author (MR) will draft the explanation and/or example for each reporting item. This will ensure that each reporting item is clear to participants in the Delphi survey.

For peer review only

All members of the Steering Committee will review and approve this preliminary list of reporting items and explanations/examples before the Delphi survey begins.

Stage 2: Delphi survey

Design

The Delphi process will consist of two rounds of electronic-based survey, response and feedback. The first round survey will include scoring of reporting guideline items from the preliminary list formed at Stage 1 and will invite additional items not included in this list. A second round survey will then be undertaken providing feedback from the previous round and inviting participants to re-score these items. Any additional reporting items identified by participants in the first round will be included for scoring by participants in the second round of the Delphi process.

Participants

We will invite three groups of stakeholders to participate in the Delphi survey. Stakeholder groups will be chosen to encompass all aspects of pharmacogenetic research.

1. Those who undertake primary pharmacogenetic research

We will ask members of pharmacogenetics networks, such as the Pharmacogenomics Research Network (PGRN) and the UK Pharmacogenetics and Stratified Medicine Network, to participate in our Delphi survey. We will perform searches to identify these networks and contact experts in the field to ensure that all major networks across the globe are identified.

2. Those who systematically review pharmacogenetic research data

We will email the contact authors of systematic reviews of pharmacogenetics studies identified by searching PubMed, using appropriate search terms such as “pharmacogenetics”,

For peer review only

“pharmacogenomics”, “systematic review” and “meta-analysis”. We will consult an information specialist (Eleanor Kotas) to design the search strategy). We will ask these authors to complete the survey if they participated in the data extraction and/or data synthesis of the review, and we will also ask this author to forward the survey on to other authors who participated in the data extraction and/or data synthesis.

3. Those who publish pharmacogenetic research

We will identify these individuals by contacting the editors-in-chief of key pharmacogenetics journals. We will perform searches to identify key journals, using search terms “pharmacogenetics”, “pharmacogenomics”, “precision medicine”, “personalised/personalized medicine”. We will also consider journals listed on the “The SCImago Journal & Country Rank” portal[5] under the subject category “Genetics”. We will contact experts in the field to ensure that we have not missed any key journals. We will ask the editors-in-chief to participate in the survey themselves, and also to forward the survey on to editors at their journal.

The decision of how many individuals to invite to complete a Delphi survey is not based on statistical power, and often must be a pragmatic choice.[6] Generally, there should be good representation of experts from the key stakeholder groups, who have a deep understanding of the relevant issues. Our aim is to maximise the number of participants who complete the Delphi survey, to ensure that the total number of participants within each stakeholder group is sufficiently large to yield a meaningful statistical analysis.

For the stakeholder group of primary researchers, we are confident that a sufficiently large number of individuals will complete the survey; two of the networks (the PGRN network and the UK Pharmacogenetics and Stratified Medicine Network) whose members we will invite to complete the survey together have over 1000 members. For the stakeholder groups of systematic reviewers and

For peer review only

journal editors, we hope that the snowballing technique i.e. asking review authors and editors-in-chief to forward the survey on to other individuals, will ensure that the numbers of participants in these groups is sufficiently large.

Recruitment process and ethical considerations

We will e-mail the individuals listed above with information about the STROPS project and the Delphi process and an invitation to complete Round 1 of the Delphi survey within three weeks (see Supplementary File 1). We will inform invitees that participation in the survey is optional, and that we will assume informed consent if an invitee responds to Round 1 of the survey. We will inform invitees that all data will be anonymised; we will allocate a unique identification number to each participant in the Delphi survey. When registering for the study on the survey website, participants will also be asked to tick a box if they agree to participate in the study.

We will send a reminder e-mail at the end of the second week to prompt completion of the survey. We will not be able to send a reminder e-mail to individuals who received forwarded invitations, as we will not have contact information for these individuals at this stage. All participants who complete the first round of the Delphi survey will be invited to participate in the second round. However, we will inform invitees that completion in the first round does not necessitate completion in the second round, and we will remind participants of the first round that this is the case, when we invite them to complete the second round.

If attrition rates (the degree of non-response to the second round of the survey) are high, either for a particular stakeholder group or overall, then we will adopt strategies to increase response rates. Generally, a response rate of around 80% for the second round of a Delphi survey can be considered satisfactory in most scenarios.[6] Strategies for increasing response rates to Round 2 may include:

For peer review only

sending personalised reminder e-mails, offering acknowledgement in the published reporting guideline, and extending the period of time for which the second round is open.

If participants that do not respond to Round 2 have different opinions to participants from the same stakeholder group who complete both rounds, then attrition bias has occurred and this will affect the results of the Delphi survey. If response rates to Round 2 are less than 80%, we will investigate the risk of attrition bias. We will calculate average Round 1 scores for each participant, and then plot these scores according to whether participants completed Round 2 or not for each stakeholder group. We will visually examine these plots to assess the likelihood of attrition bias.

Participant characteristics

We will ask participants to provide their name, e-mail address and their consent to be acknowledged as a participant in the Delphi survey in publications arising from this project. Demographic data regarding the participant's profession and previous involvement with reporting guideline development will be collected; all demographic data will be anonymised.

Delphi scoring

Participants will be asked to score each of the reporting guideline items listed using a scale of 1 to 9, with 1 to 3 labelled 'not important for inclusion in the guideline', 4 to 6 labelled 'important but not critical for inclusion in the guideline' and 7 to 9 labelled 'critical for inclusion into the guideline'.[7] Participants will also be given the option to score a reporting guideline item as 'unable to score' if they are unable to offer an opinion as to whether the item is important or not.

Software

For peer review only

The Delphi survey will be conducted using DelphiManager, a web-based system designed by the COMET Initiative (http://www.comet-initiative.org/delphimanager/) to facilitate the building and management of Delphi surveys.

Delphi Round 1

Reporting guideline items will be presented in the order in which they would be addressed in the pharmacogenetic study report and will be grouped under relevant headings (i.e. title and abstract, introduction, methods, results, discussion and other information). Participants will be asked to score each item as described previously. Participants will also be given the chance to add items that they believe should be included in the reporting guideline.

Round 1 analysis

For each item, the number of participants who have scored the item and the distribution of scores will be summarised. Participants who scored an item as “unable to score” will be excluded from the analysis for that particular item. We will review all additional guideline reporting items listed by participants to ensure that they are not covered by the existing list of reporting guideline items. Additional reporting guidelines items that are not already covered will undergo formal review and discussion by the Steering Committee, and if appropriate, will be added to the list of reporting guideline items presented in Round 2.

Delphi Round 2

In Round 2, each participant who participated in Round 1 will be shown the number of respondents and distribution of scores for each item from Round 1, for each stakeholder group separately. Participants will also be reminded how they personally scored each item in Round 1. Participants will be asked to consider the responses from other Delphi participants, and to re-score the items.

For peer review only

In addition, if a participant changes their score from 'not critical' in Round 1 to 'critical' in Round 2 or from 'critical' in Round 1 to 'not critical' in Round 2, they will be asked to provide their reasoning for this change.

Additional items identified as part of Round 1 will be scored by participants in Round 2.

Round 2 analysis

For each item, the number of respondents and the distribution of scores will be summarised. Participants who scored an item as "unable to score" will be excluded from the analysis for that particular item. We will assess the possibility of attrition bias occurring by comparing item scores from participants who completed Round 1 only, to item scores from participants who completed both Round 1 and Round 2. We will also examine changes in scores between rounds and summarise the reasons given for changes from 'critical' to 'non-critical' and vice versa.

Consensus definition

Guideline reporting items will be prioritised if at least 70% of participants score them as 'critical'. The rationale for this threshold is that consensus that an item ought to be included in the reporting guideline requires agreement by the majority regarding the critical importance of the outcome. This threshold for consensus has been used previously in the development of the COS-STAR reporting guideline [8].

Stage 3: Consensus meeting

The Steering Committee and one or two representatives from each stakeholder group will meet to consider the results of the Delphi survey and to finalise the list of items for the draft reporting guideline. Representatives from each stakeholder group must have completed both rounds of the

For peer review only

Delphi survey. Furthermore, we aim to include at least one non-UK representative from each stakeholder group in the consensus meeting. The meeting will be conducted via conference call.

At the meeting, MR will present a summary of the results of how each stakeholder group had scored each reporting guideline item (from Stage 2), and the number of stakeholder groups who achieved consensus. Meeting attendees will discuss each reporting guideline item in turn, and will make a decision on whether to include the item in the reporting guideline or not. Items that reached consensus from all stakeholder groups in the Delphi survey will be considered first. Each remaining item will then be considered in turn according to the number of stakeholder groups where consensus was achieved, i.e. the next batch of items to be considered will be those that reached consensus in all but one stakeholder group.

Stage 4: Development and publication of reporting guideline and explanatory document

We will draft the initial reporting guideline and the explanatory document concurrently. The purpose of the explanatory document is to provide the meaning and rationale for each reporting item alongside examples of good reporting practice. For each item, we will also document the origin of the item (Steering Committee or Delphi participants) and the degree of consensus achieved from the Delphi survey.

We will pilot-test the draft reporting guideline with researchers who are yet to publish the findings of their pharmacogenetics study, and with researchers who have already published a pharmacogenetics study. We will identify these individuals by contacting pharmacogenetics researchers based at the University of Liverpool, and by contacting authors of published pharmacogenetics studies listed on PubMed. We will incorporate feedback on the content, format, and usefulness of the guideline from these researchers in the final reporting guideline.

For peer review only

Stage 5: Dissemination of the reporting guideline

Dissemination activities will include presenting the final reporting guideline at conferences relevant to pharmacogenetics research.

For peer review only

PATIENT AND PUBLIC INVOLVEMENT STATEMENT

The STROPS guideline will be developed without patient involvement. Patients were not invited to contribute to the writing or editing of this document for readability or accuracy.

ETHICS

The University of Liverpool Ethics Committee has been consulted and confirmed ethical approval for this study (Reference: 3586).

DISCUSSION

We plan to conduct our project using robust methodology for developing reporting guidelines proposed by the EQUATOR network.[2] Using such methodology will ensure that the resulting reporting guideline is useful and widely disseminated. The EQUATOR approach includes a face-to-face consensus meeting, which follows the Delphi survey. This meeting often involves the steering group and a selection of stakeholders who took part in the Delphi survey. Simera et al.[9] conducted a survey of authors of 37 reporting guidelines and reported that the median number of people participating in consensus meetings for these reporting guidelines was 22. Due to a lack of funding for this project to cover travel and accommodation costs, we will be unable to arrange a face-to-face consensus meeting including this number of participants. Our consensus meeting will only involve the members of the Steering Committee (n=6) and one or two representatives of the key stakeholder groups, and the meeting will be conducted via conference call. We will invite a large, international and multidisciplinary cohort to participate in the Delphi survey, so that meeting attendees are able to base their decisions on the opinions of this wider cohort.

We will prioritise items for inclusion in the guideline if at least 70% of participants score them as 'critical'. Although the choice of this threshold is somewhat subjective, pre-specification of the

For peer review only

threshold in this protocol ought to provide assurance that we will not define consensus in a post-hoc way, and therefore our own opinions will not bias the results of the Delphi survey [10].

There is currently no guideline for the reporting of pharmacogenetic studies that has been developed using a widely-accepted robust methodology. The final guideline will not only improve the transparency of reporting of pharmacogenetic studies, but will also facilitate the conduct of high-quality systematic reviews and meta-analyses, and thus improve the power to detect genetic associations. With the increasing number of meta-analyses of pharmacogenetic studies that are being undertaken, it is important that reporting of key data in study reports is improved in order to allow robust synthesis of the studies.

REFERENCES

1. Little J, Higgins J, editors. The HuGENet™ HuGE review handbook, version 1.0: Ottawa, Ontario, Canada: Department of Epidemiology and Community Medicine, Faculty of Medicine, University of Ottawa; 2006 [updated 23 October 2015; cited 2017 1 November]. Available from: http://www.medicine.uottawa.ca/public-health-genomics/web/assets/documents/HuGE_Review_Handbook_V1_0.pdf accessed 1 November 2017.
2. Moher D, Schulz KF, Simera I, et al. Guidance for developers of health research reporting guidelines. *PLoS Med* 2010;7(2):e1000217.
3. UK EQUATOR Centre. Equator Network 2018 [Available from: https://www.equator-network.org/ accessed 25th Feb 2019].
4. Jorgensen AL, Williamson PR. Methodological quality of pharmacogenetic studies: issues of concern. *Stat Med* 2008;27(30):6547-69.
5. SCImago. SJR - SCImago Journal & Country Rank [Portal] (N.d.) [cited 29th May 2019. Available from: http://www.scimagojr.com.
6. Williamson PR, Altman DG, Bagley H, et al. The COMET handbook: version 1.0. *Trials* 2017;18(3):280.
7. Guyatt GH, Oxman AD, Kunz R, et al. GRADE guidelines: 2. Framing the question and deciding on important outcomes. *J Clin Epidemiol* 2011;64(4):395-400.
8. Kirkham JJ, Gorst S, Altman DG, et al. Core outcome set–STAndards for reporting: the COS-STAR statement. *PLoS medicine* 2016;13(10):e1002148.
9. Simera I, Altman DG, Moher D, et al. Guidelines for reporting health research: the EQUATOR network's survey of guideline authors. *PLoS Med* 2008;5(6):e139.
10. Williamson PR, Altman DG, Blazeby JM, et al. Developing core outcome sets for clinical trials: issues to consider. *Trials* 2012;13(1):132.

AUTHOR STATEMENT

For peer review only

MR, ALJ, JK and KD conceived the idea for the project. All authors (MR, ALJ, JK, KD, GD and DJS) contributed to the design of the study. MR is the guarantor, and drafted the manuscript. All authors (MR, ALJ, JK, KD, GD and DJS) read, provided feedback and approved the final manuscript.

FUNDING

MR is supported partly by Liverpool Reviews and Implementation Group (LR*i*G, based at the University of Liverpool), based on funding from the National Institute for Health Research Health Technology Assessment Programme (URL, http://www.nets.nihr.ac.uk/programmes/hta), and partly by the Research, Evidence and Development Initiative (READ-It) project. READ-It (project number 300342-104) is funded by UK aid from the UK government; however, the views expressed do not necessarily reflect the UK government's official policies.

CONFLICTS OF INTERESTS

The authors declare that they have no conflicts of interests.

For peer review only

Supplementary file 1: Initial invitation e-mail

Dear NAME,

We are a team of researchers based in different institutions across the UK (University of Liverpool; University of St. Andrews; Cochrane Editorial Unit, London), and we are currently developing the STrengthening the Reporting Of Pharmacogenetic Studies (STROPS) guideline. We are conducting a Delphi survey to gain consensus opinion on which reporting items ought to be included in this guideline.

We would like to invite you to be part of the development of this guideline by completing our Delphi survey.

The Delphi survey will consist of two rounds of electronic-based survey, response and feedback. Participants will be asked to score reporting items based on their opinions about the importance of these items for inclusion in the reporting guideline. All scoring data will be anonymised. Participation is of course optional, and furthermore, completion of the first round survey does not necessitate completion in the second round survey. We will assume informed consent if we receive a response to the first round of the Delphi survey.

If you would like to participate in the development of this reporting guideline, please complete the first round of the Delphi survey by DATE using the following link: LINK

Kind Regards,
Marty Richardson

On behalf of the Steering Committee of the STROPS (STrengthening the Reporting Of Pharmacogenetic Studies) project

For peer review only
